# Migrant workers’ perception and awareness of health insurance coverage in Brunei Darussalam

**DOI:** 10.1186/s12913-024-10623-x

**Published:** 2024-08-19

**Authors:** Alice Lai, Noorfaizah Mohammad, Ashish Trivedi, Zaidah Murang, Nik Tuah

**Affiliations:** 1 Occupational Health Division, Ministry of Health, Bandar Seri Begawan, Brunei Darussalam; 2 PAPRSB Institute of Health Sciences, Universiti Brunei Darussalam, Bandar Seri Begawan, Brunei Darussalam; 3 Department of Primary Care and Public Health, Imperial College London, London, UK

**Keywords:** Migrant worker, Migrant health, Health insurance, Awareness, Perception, Health coverage

## Abstract

**Background:**

Migrant workers are recognized as a vulnerable group of population in the context of accessibility to healthcare services as they are likely to experience multiple challenges and barriers. This study aimed to assess the awareness and perceived knowledge on health insurance coverage among documented migrant workers in Brunei Darussalam.

**Methods:**

This cross-sectional, interviewer-administered study used a pre-designed questionnaire on migrant workers attending the Foreign Worker Health Screening Centre from June until September 2019. Data were analyzed for association between perceived insurance status on health-seeking behavior.

**Results:**

The study obtained responses from 469 documented migrant workers (93.8%). 75.1% reported being aware of and having health insurance coverage; and of these, 57.1% were aware of the type of health insurance cover they had. 45.5% and 50.6% had poor knowledge whether their health insurance covered for hospitalization or outpatient expenses, respectively. No significant association was found between the migrant workers’ perceived status of insurance and not seeking medical care due to financial barriers (*p* > 0.05).

**Conclusions:**

A high proportion of documented migrant workers in Brunei Darussalam reported knowledge of having health insurance; however, there was lack of awareness on its actual coverage. By including migrants’ health in a nation’s healthcare governance, the health rights of migrant workers can be addressed thus aiming to achieve universal health coverage for all individuals.

## Introduction

The International Labour Organisation (ILO) reported that in 2018 there were 164 million migrant workers worldwide, with the majority living in 3 sub-regions: North America; Northern, Western and Southern Europe; and Arab countries [1, 2]. In the present era, almost all countries are affected by international migration which has become an inherent element of globalization, thus raising the issues of human protection and health crisis among migrant workers and their dependents [3]. The health of an individual is not solely influenced by its biological characteristics but also socio-demographic characteristics that comprise earnings, housing, work conditions and education; all of which are significant predictors of migrant workers being able to access healthcare services [4]. Migrant workers are recognized to be a vulnerable population as they are susceptible to multiple challenges and barriers, particularly when accessing healthcare services. They are also mostly exposed to poor work conditions and occupational health hazards, tend to earn low wages whilst working for longer hours, and are often subject to human rights violation, abuse and violence [5, 6]. The migrant population has become an underprivileged group particularly in developed countries as they lack social support hence suffering from work-related diseases that may go unnoticed [7].

Participation in health insurance schemes and its awareness among migrant workers can be of vital importance in enhancing their access to essential healthcare and achieving universal health coverage [8–10]. Health insurance helps cover for medical and surgical expenses. Workers’ health insurance is commonly purchased by employer or self-bought or financed through government insurance policies. Having a health insurance has been associated with better health outcomes as it improves access to healthcare services, whereas uninsured individuals tend to encounter a negative reception in their attempt to access healthcare services at health facilities due to inability to settle medical bills [11–13]. However, lack of awareness regarding health insurance and its coverage can increase the vulnerability of migrant workers whilst working and residing in the host country [14, 15].

According to a report from the Labour Force Survey, the total number of migrant workers in Brunei increased from 47,500 persons in 2017 [16] to 56,600 persons in 2018 [17], representing a growth rate of 7.2%. By 2018, the migrant worker population made up 25.6% of Brunei’s workforce. Healthcare in Brunei Darussalam is readily available and accessible to all residents through primary and tertiary care centres in all four districts of the country. Public healthcare is financed by the government and is free for Brunei citizens, permanent residents, and expatriate government employees (including their dependents), while non-citizens including private sector expatriates and migrant workers mainly pay out of pocket or are covered by health insurance premiums purchased by their employer [18, 19]. National legislations require that all registered migrant workers have a medical insurance and worker insurance. These insurances are to cover for basic healthcare services for outpatient visits and hospitalizations, work-related injuries and occupational fatalities, and to cover for the cost of body repatriation in the event of a fatality or demise. Employers are responsible to provide insurance coverage for the health and safety protection of migrant workers [20]. Despite the growing number of migrant workers in the country in the last few years due to diversification of industries that saw an increase in employment of professionals and labourers, very little is known about awareness and perceived knowledge amongst migrant workers on their health insurance coverage and access to healthcare services in the country.

The aim of this study was to assess the knowledge and awareness of documented migrant workers in Brunei on their health insurance coverage. The objectives set out were to describe the sociodemographic characteristics, estimate the coverage of health insurance, and analyze for association between awareness of health insurance status and health-seeking behavior.

## Methods

This cross-sectional study used systematic random sampling of attendees at the Foreign Worker Health Screening Centre (FWHSC), Ministry of Health over a four-month period from June until September in 2019. The FWHSC received documented migrant workers who attended for health screening as part of the process prior to obtaining their Employment Pass. The selection of participants was based on even numbering of queue tickets of attendees to the FWHSC. After calculating for ideal power and sample size, 500 participants were invited to the study. The inclusion criteria were documented migrants who were employed in the private sector in Brunei, aged 18–60, had work experience in the country for at least one year. Undocumented migrants and those below age 18 and over 60 years were excluded from the study.

The study used a pre-designed and interviewer-administered questionnaire adapted from a similar study [21], which was modified to suit the local setting. The final questionnaire was pretested for reliability and clarity before being used for the study. The survey questionnaire collected data on sociodemographic profile (age, gender, nationality, education, occupation, working hours, salary, and duration of employment in Brunei), and knowledge and awareness of participants’ health insurance coverage.

Data were analyzed using R statistical software (version 3.4.1) and presented as descriptive statistics using frequency and percentage. Pearson’s chi-square and Fisher’s exact tests were used for comparison of categorical variables and *p* < 0.05 was considered as statistically significant.

The study was given approval by PAPRSB Institute of Health Sciences Research Ethics Committee (IHSREC) (UBD/PAPRSBIHSREC/2019/7).

## Results

Table 1 a total of 469 migrant worker participants met the inclusion criteria and completed the questionnaire, whilst 31 were excluded as they were completed by employment agents/ employers who had accompanied their migrant worker employee to the FWHSC. Majority of respondents were male (68.9%), from Indonesia (42.9%) and the Philippines (23.5%).

**Table 1:** Socio-demographic and work characteristics of migrant worker study population

Socio-demographic variables	Number (*N* = 469)	Percentage (%)
Age group (in years)		
≤ 20	3	0.6
21–30	112	24.0
31–40	177	37.8
41–50	144	30.6
> 50	33	7.0
Mean age (± SD)	37.6(± 8.70)	
Gender		
Male	329	70.2
Female	140	29.8
Nationality		
Indonesian	202	43.0
Filipino	106	22.6
Malaysian	53	11.4
Bangladeshi	50	10.6
Indian	46	9.8
Other	12	2.6
Marital status		
Single	136	29.0
Married	319	68.0
Other	14	3.0
Number of dependents (family)		
0	415	88.5
1–3	48	10.2
4–6	5	1.0
≥ 7	1	0.2
Highest level of education		
Primary	85	18.2
Secondary	254	54.0
Diploma	56	12.0
Undergraduate and/or higher	69	14.0
Other	5	1.0
Number of dependents (family)		
None	415	88.5
1–3	48	10.2
4–6	5	1.0
≥ 7	1	0.2
Industry^a^		
Construction	92	19.6
Hotels and Restaurants	82	17.4
Wholesale and retail trade; repair of motor vehicles and motorcycles	81	17.2
Domestic worker	64	13.6
Manufacturing	57	12.2
Agriculture	15	3.2
Oil and gas	2	0.4
Other	76	16.2
Monthly salary (in BND$)		
< 750	327	69.8
≥ 750	142	30.2
Employment characteristics	Mean (± SD)	
Working hours per day (6-days work week)	9.43 (± 2.21)	
Number of off-days per month	3.01 (± 1.91)	
Number of years in Brunei	9.27 (± 7.69)	

^a^Type of industry is categorized according to the United Nations International Standard Industrial Classification of All Economic Activities (ISIC) Rev 4

Table 2 shows that 75.1% of the migrant workers perceived that they had health insurance coverage. Of this group, 57.1% knew what type of insurance they had whilst 42.9% were uncertain. 43.5% of workers reported having health insurance coverage for the whole duration of their two-year employment contract. A significant percentage of insured migrant workers had poor knowledge whether their insurance covered for hospitalization expenses (45.5%) or outpatient expenses (50.6%). Majority of participants (68.9%) perceived that in the event of a hospitalization, their employer would cover their medical expenses if it was work-related. 45.2% reported that they would have to self-pay if their medical expenses were incurred from a non-work related injury. Only 10% and 5.3% of insured migrant workers believed that their insurance played a role in financing their work-related injury and non-work related injury hospitalization expenses, respectively.

**Table 2:** Awareness of health insurance coverage among migrant worker study population

Variable	Number (*N* = 469)	Percentage (%)
Have health insurance		
Yes	352	75.1
No	117	24.9
If Yes (*n* = 352):		
Type of health insurance		
Medical insurance	92	26.1
Worker insurance	109	31.0
Unsure	151	42.9
Duration of insurance coverage (in years)		
1	94	26.7
2	153	43.5
Unsure	105	29.8
Insurance covers hospitalization expenses		
Yes	163	46.3
No	29	8.2
Unsure	160	45.5
Insurance covers outpatient expenses		
Yes	111	31.5
No	63	17.9
Unsure	178	50.6
Who pays for work-related injury hospitalization		
Self only	40	8.5
Employer	323	68.9
Insurance Company	47	10.0
Unsure	59	12.6
Who pays for non-work related injury hospitalization		
Self only	212	45.2
Employer	143	30.5
Insurance Company	25	5.3
Unsure	89	19.0

Tables 3, 4 and 5 present results from Fisher’s Exact and Chi-square tests which looked for association between perceived status of health insurance and health-seeking behavior among documented migrant workers. There was no significant association for perceived health insurance status and visit to a healthcare facility in the last year (*p* = 0.50), nor with not seeking medical care because of financial reasons (*p* = 0.95). There was also no significant association between perceived health insurance coverage and nationality nor level of education (*p* = 0.21 and *p* = 0.15 respectively).

**Table 3:** Health-seeking behavior and its relation with perceived health insurance status

Variable	Number	Percentage (%)
Number of visits to healthcare facilities in the past one year		
0	284	60.6
1–3	181	38.6
≥ 4	4	0.9
Not seeking medical care because of cost		
Yes	19	4.0
No	450	96.0

**Table 4:** Association between perceived health insurance status and health-seeking behavior

**Perceived health insurance status**	**Visit to a health facility in last one year**		**Number**	***P*-value**
	**No visit**	**One or more visit**		
Have health insurance	215 (59.7%)	145 (40. 3%)	360	0.50
No health insurance	69 (63.3%)	40 (36.7%)	109	
**Perceived health insurance status**	**Did not seek medical care because of costs**		**Number**	***P*-value**
	**Yes**	**No**		
Have health insurance	14 (4.0%)	334 (96.0%)	348	0.95
No health insurance	5 (4.1%)	116 (95.9%)	121

**Table 5:** Association between nationality and educational status with perceived health insurance status

**Nationality (*n* = 457)**	**Have insurance**	**Unaware of having insurance**	**Number**	***P*-value**
Indonesia	156 (77.3%)	46 (22.7%)	202	
Philippines	74 (69.8%)	32 (30.2%)	106	
Malaysia	46 (86.8%)	7 (13.2%)	53	0.21
Bangladesh	38 (76%)	12 (24%)	50	
India	35 (76.1%)	11 (23.9%)	46	
**Highest Education (*n* = 464)**	**Have insurance**	**Unaware of having insurance**	**Number**	***P*-value**
Primary	58 (68.2%)	27 (31.8%)	85	
Secondary	196 (77.2%)	58 (22.8%)	254	0.15
Diploma	47 (83.9%)	9 (16.1%)	56	
Undergraduate or higher	50 (72.5%)	19 (27.5%)	69	

## Discussion

Major factors that are known to contribute to poor health outcomes among the migrant population are sub-optimal working and living conditions, lack of access to health services mainly due to financial constraints, and lack of awareness due to linguistic and cultural barriers [22]. Health insurance is a significant constituent of a nation’s social security system. It is regarded as one of the most fundamental instruments to obtain adequate accessibility to medical support [23]. Optimum coverage of migrant workers with health insurance can improve their access to healthcare services [8, 24]. In this study, a substantial number of migrant workers (75.1%) reported that they were covered by insurance for health purposes. This was higher than those reported in regional countries such as Singapore (61%), Thailand (58.9%) and Malaysia (50%) [21, 25, 26]. Our study finding was likely to be attributed to the implementation of a policy by the Ministry of Home Affairs, Brunei Darussalam whereby employers were required to provide health insurance and worker insurance for registered migrant workers for the duration of their employment contract in the country (typically for two years) [20]. A similar policy was introduced by the Ministry of Manpower, Singapore in 2008 which resulted in more than 50% of their migrant worker population having medical insurance [21]. Similarly, another study in Thailand revealed that the status of health insurance among 3.5 million migrant workers escalated from 9% in 2011 to 33.7% after the introduction of a migrant health insurance policy by the Thai Ministry of Public Health [27]. In contrast to migrant workers in Asian studies, majority of migrants in the United States, particularly Hispanics, were found to have no insurance (79%) because of high costs of health insurance coverage [28]. Another US study reported that non-citizens and their children (compared to citizens) had worse access to regular ambulatory and emergency care, even when insured. Immigration status was viewed an important component of racial and ethnic disparities in insurance coverage and access to care [29].

Our study findings of almost half of the study population having poor knowledge regarding their insurance type resonates other studies [21, 30]. Some of the reasons for this were inadequate information on insurance policies given by employers to migrant worker employees, and employers themselves having poor understanding on the provisions of insurance policies and language barrier [21, 27, 31].

This study found no statistically significant association between migrant workers’ perceived status of insurance and not seeking medical care due to financial barrier. Similarly, there was no significant association between perceived health insurance status and nationality nor education level. This unawareness may be due to poor information from employers or language barrier, which were also observed in other regional countries [21, 27, 31]. Majority (68.9%) were of the impression that in the event of a work-related hospitalization, their employer would be responsible for their medical expenses. According to Brunei’s Employment Order 2009 and Workplace Safety and Health Order 2009, it is the duty of every employer to ensure their employee’s health, safety and welfare at the workplace, including timely payment of salary, covering medical expenses and body repatriation expenses in the event of a fatal death [32]. It can be postulated that migrant workers in Brunei Darussalam, regardless of their perceived insurance status, do not experience financial difficulty when accessing healthcare services due to the provisions in national legislations. This is in contrast to other studies that reported financial constraint as a significant barrier for migrant workers accessing healthcare services [29, 30].

There were some challenges encountered during the conduct of this study. Though the response received for participation was encouraging, language barrier was viewed as a barrier as many migrant workers, although having worked and lived in Brunei Darussalam for at least two years, did not speak the national language (Malay) or spoke very little English. As resources for this study were limited, no professional translators were available.

## Conclusions

This study observed that a high proportion of documented migrant workers in Brunei Darussalam were of the knowledge that they had health insurance coverage. However, they lacked awareness on the type and provisions under their insurance coverage. There was also no significant association between migrant workers of different nationalities and educational status with their perceived insurance status. Universal health coverage which includes provision of health insurance to the migrant population and improvement of awareness of this among migrant workers can improve their access to healthcare thus closing the gap towards achieving universal health coverage. This study identifies considerations for future policy-making in this area, and provides an impetus for future research such as further exploring health-seeking behavior in relation to barriers to healthcare access among migrant workers, including their dependents. The findings can be used to facilitate discussions at various levels of policy-making, such as having migrant workers be present during explanation of their health insurance coverage and in their own language.

## Data Availability

The datasets generated and analyzed during the current study are not publicly available due to confidentiality policies, but are available from the corresponding author on reasonable request.
